# Proteomic changes during experimental de- and remyelination in the corpus callosum

**DOI:** 10.1371/journal.pone.0230249

**Published:** 2020-04-09

**Authors:** Gabor T. Szilagyi, Arkadiusz M. Nawrocki, Krisztian Eros, Janos Schmidt, Katalin Fekete, Maria L. Elkjaer, Kirsten H. Hyrlov, Martin R. Larsen, Zsolt Illes, Ferenc Gallyas

**Affiliations:** 1 Department of Biochemistry and Medical Chemistry, University of Pécs Medical School, Pécs, Hungary; 2 Department of Biochemistry and Molecular Biology, University of Southern Denmark, Odense, Denmark; 3 Szentagothai Research Centre, University of Pécs, Pécs, Hungary; 4 Nuclear-Mitochondrial Interactions Research Group, Hungarian Academy of Sciences, Budapest, Hungary; 5 Department of Neurology, Odense University Hospital, Odense, Denmark; 6 Institute of Clinical Research, BRIDGE University of Southern Denmark, Odense, Denmark; 7 Institute of Molecular Medicine, University of Southern Denmark, Odense, Denmark; Heinrich-Heine-Universitat Dusseldorf, GERMANY

## Abstract

**Background:**

In the cuprizone model of multiple sclerosis, de- and remyelination can be studied without major interference from the adaptive immune responses. Since previous proteomic studies did not focus on the corpus callosum, where cuprizone causes the most pronounced demyelination, we performed a bottom up proteomic analysis on this brain region.

**Methods:**

Eight week-old mice treated with 0.2% cuprizone, for 4 weeks and controls (C) were sacrificed after termination of the treatment (4wD), and 2 (2dR) or 14 (2wR) days later. Homogenates of dissected corpus callosum were analysed by quantitative proteomics. For data processing, clustering, gene ontology analysis, and regulatory network prediction, we used Perseus, PANTHER and Ingenuity Pathway Analysis softwares, respectively.

**Results:**

We identified 4886 unmodified, single- or multi phosphorylated and/or gycosylated (PTM) proteins. Out of them, 191 proteins were differentially regulated in at least one experimental group. We found 57 proteins specific for demyelination, 27 for early- and 57 for late remyelinationwhile 36 proteins were affected in two, and 23 proteins in all three groups. Phosphorylation represented 92% of the post translational modifications among differentially regulated modified (PTM) proteins with decreased level, while it was only 30% of the PTM proteins with increased level. Gene ontology analysis could not classify the demyelination specific proteins into any biological process category, while allocated the remyelination specific ones to nervous system development and myelination as the most specific subcategory. We also identified a protein network in experimental remyelination, and the gene orthologues of the network were differentially expressed in remyelinating multiple sclerosis brain lesions consistent with an early remyelination pattern.

**Conclusion:**

Proteomic analysis seems more informative for remyelination than demyelination in the cuprizone model.

## Introduction

Multiple sclerosis is the most common chronic inflammatory demyelinating disease that affects mainly young adults [1]. The disease is progressive, and impacts the central nervous system with a complex pathomechanism involving both neurodegenerative and inflammatory characteristics [1]. The current disease modifying therapies aim to prevent relapses by suppressing inflammation in the relapsing–remitting form of the disease, but limited options are available to prevent demyelination or axonal degeneration [2]. Therefore, intensive research is going on to identify novel therapeutic targets.

The complexity and heterogeneity of multiple sclerosis pathology cannot be replicated by a single animal model; the most commonly used experimental autoimmune encephalomyelitis, and toxin- and/or virus-induced demyelination models capture only certain clinical and pathological features of the disease. The neurotoxin cuprizone (bis-cyclohexanoneoxalyldihydrazone, CPZ) causes reproducible, anatomically selective and reversible demyelination [3] that is not affected by the absence of T and B cells while the blood–brain barrier is considered to be intact [4]. Therefore, and in contrast to other multiple sclerosis models, de- and remyelination can be studied without interference from the contribution of adaptive immune responses [5].

Proteomic approach was successfully applied for studying pathomechanism [6, 7] of or finding new drug targets [8, 9] for various diseases. Interestingly, we found only three previous studies utilizing this approach for analysing CPZ-induced reversible demyelination [10–12]. None of them measured proteomic changes in the corpus callosum, where CPZ-induces the most pronounced demyelination [13]. Accordingly, in the present study, we assessed proteomic changes during de- and remyelination in the corpus callosum of CPZ treated mice.

## Materials and methods

### Materials

Cuprizone (CPZ) was from Sigma-Aldrich (Budapest, Hungary) The Protease inhibitor mix without EDTA and PhosSTOP phosphatase inhibitor cocktail were from Roche Applied Science (Meylan, France). Benzonase was from Merck (Darmstadt, Germany). Lysyl endopeptidase (Lys-C) was from Wako Pure Chemical Industries (Osaka, Japan). Modified trypsin was from Promega (Madison, WI, USA). iTRAQ 4-plexTM was from Applied Biosystem (Foster City, CA, USA). Titanium dioxide beads were from GL Science (Japan). Poros Oligo R3 reversed phase chromatographic materials were from Applied Biosystems (Framingham, MA, USA). PHOS-select^TM^ metal chelate beads were from Sigma-Aldrich (St. Louis, MO, USA). TSK amide-80 HILIC 3 μm from Tosoh Bioscience (Stuttgart, Germany). 3M Empore C8 disk was from 3M Bioanalytical Technologies (St. Paul, MN, USA). All other reagents used in the experiments were of sequencing grade, and the water was from a Milli-Q system (Millipore, Bedford, MA).

### Ethic statement and cuprizone treatment

The animal experiments were performed according to the Guide for the Care and Use of Laboratory Animals published by the US National Institutes of Health, and the protocol was approved by the Animal Research Review Committee, University of Pecs, Hungary. All animal experiments were controlled by trained personnel, and all efforts were made to minimize animal suffering.

C57BL/6 male mice were purchased from Charles River, Innovo Kft (Isaszeg, Hungary) and kept under standardized circumstances (controlled temperature, humidity and 12:12 h light-dark cycles.) Food and water were freely available.

Starting at 8 weeks of age, 20 animals were randomly assigned into 4 groups. Three groups were nourished with powdered rodent chow (1324 Altromin, Germany) containing 0.2% CPZ for 4 weeks ad libitum to induce demyelination, as described previously [14]. Control (C) group received the same without CPZ. To follow the methodical effect of CPZ treatment, the weights of the mice were measured twice a week [14], extent of demyelination was assessed by MRI imaging [15] at the end of CPZ treatment and before sacrificing the animals. The mice were sacrificed with cervical dislocation in deep isoflurane anaesthesia after termination of the treatment (4wD), and 2 (2dR) or 14 (2wR) days later. Brains were excised and the corpus callosums were dissected and snap-frozen on dry-ice. The samples were stored at −80°C until further processing.

### Sample preparation

Dissected corpus callosums were homogenised in buffered solution (pH 7.5) consisting of protease and phosphatase inhibitors, 10mM DTT, 10mM sodium orthovanadate and benzonase (0.05%) using Dounce homogenizer, followed by probe sonication. Proteins were collected by centrifugation at 20000g after being precipitated by addition of 8 volumes of -20°C acetone and 1 volume of trichloroacetic acid. Protein pellets were solubilized with 6 M urea, 2 M thiourea, 10 mM DTT, followed by alkylating with iodoacetamide and digesting with lysyl endopeptidase and trypsin. Peptides were purified using homemade RP columns (both C8 and C18 resins were used) and labelled by isobaric tags (iTRAQ 4-plex^TM^**)** for relative quantitation. Based on amino acid composition analysis, equal amounts of samples were labelled as follow: Control, iTRAQ-114; 4 weeks demyelination, iTRAQ-115; 2 days remyelination, iTRAQ-116; and 2 weeks remyelination, iTRAQ-117 and combined in 1:1:1:1 ratio. In order to enrich for phosphopeptides and glycosylated peptides, the TiSH (TiO_2_-SIMAC-TiO_2_) protocol [16] was applied. Prior to the nano LC-MSMS identification, the phosporylated, deglycosylated and nonmodified peptides were fractionated by HILIC as described previously [17].

### Liquid Chromatography–Mass Spectrometry (LC-MS) analysis

The peptides were separated by reverse phase chromatography using homemade C18 column (3 μm, 75 μm x 150 mm) and gradient elution at a flow rate of 250 nl/min with an aid of Thermo EASY-nLC 1000 HPLC. Approximately 1 μg of peptide sample was loaded on the column. The buffer system consisted of solvent A (aqueous formic acid solution (0.1%) and solvent B (acetonitrile/formic acid (99.9/0.1%v/v). Gradient elution varied depending on the complexity of samples and was either 60 or 130 min from 0–34% solvent B, then 5–15 min for reaching 100% B and 10 min at 100% B. Eluted peptides were analysed by MS instruments (Orbitrap Velos and Q-Exactive, ThermoFisher). For the Velos instrument: scanning range was set to 400–1500 m/z for MS^1^ scans and the first mass fixed at 110 m/z for following 7 data dependent MS^2^ scans. Peptides were fragmented by HCD at 35 NCE. For the Q-Exactive instrument: scanning range was set to 350–1600 m/z for MS^1^ scans and the first mass fixed at 110 m/z for following 12 data dependent MS^2^ scans. Peptides were fragmented by HCD at 30 NCE. Raw datafiles were processed by Thermo Proteome Discoverer software v1.4. Protein identification was carried out by searching for *Mus musculus* taxonomically restricted in the databases of the NCBI and the Swiss-Prot using Mascot V2.4.1. Search parameters were set to allow two missed cleavage site, we accepted 15 ppm mass tolerance at MS^1^ and 0.02 Da at the MS^2^ mode and we searched for variable modifications including methionine oxidation, deamidation of Asn, phosphorylation on Ser/Thr/Tyr, peptide N-terminal iTRAQ labelling and lysine iTRAQ labelling and carbamidomethylation on cysteine as a fixed modification. The relevance threshold was set to >20 MASCOT score. Data from ProteomeDiscoverer was further processed using Excel (Microsoft).

The mass spectrometry proteomics data have been deposited to the MassIVE data repository (Mass Spectrometry Interactive Virtual Environment) **ftp://MSV000083506@massive.ucsd.edu**

### Immunohistochemistry

We performed immunohistochemistry utilising protein phospho (p)-Ser, p-Thr and p-Tyr specific primary antibodies (Santa Cruz Biotechnology) on brain sections of the animals (n = 3) from all four treatment groups. Briefly, the chilled brains were removed, formalin-fixed, paraffin-embedded and sectioned (8 μm). The sections were dewaxed, exposed to 500 W microvawe for 3 x 5 min (antigene exposure) in 0.1 M citrate buffer pH 6.0, then the endogenous peroxidase activity was blocked in 0.1 M phosphate buffered saline (PBS) pH 7.4 containing 3% hydrogenperoxide for 20 min. The sections were blocked in PBS containing 0.2 10% bovine serum albumin (BSA) for 30 min, then were exposed to said primary and horseradish peroxidase conjugated anti-mouse IgG (Sigma) secondary antibodies in PBS containing 1% BSA overnight and for 1 h, respectively. Antibodies were diluted according to the manufacturer’s recommendation, and between all steps a 3 x 5 min washing in PBS was performed. Antibodies were visualised by exposing the sections to 0.067% diaminobenzidine solution in PBS containing 0.02% hydrogen peroxide for 5–15 min against negative controls lacking primary and/or secondary antibody exposure. Nuclei were counterstained by Meyer’s hematoxylin for 2 min then the sections were dehydrated and Balsam Canada (Sigma) mounted. The sections were scanned by Panoramic midi slide scanner at 1200 dpi resolution. For quantitative analysis, all nuclei and staining artefacts were eliminated from the sections and staining intensities of corpus callosums were normalised to that of left and right retrosplenial area, ventral part, layer 1 by an expert blind to the experiment by using Molecular Devices’ MetaXpress® image analyser software.

### Gene Ontology (GO), pathway and functional correlation analyses

Deregulated proteins were categorised into protein classes using Protein Analysis Through Evolutionary Relationships (PANTHER) classification system software (http://www.pantherdb.org) and the general annotation from UniProt (http://uniprot.org). For clustering, we used the Perseus software platform (http://www.perseus-framework.org) developed by the Max Planck Institute of Biochemistry (https://maxquant.net/perseus/). Gene ontology (GO) analysis of biological processes and cellular components was performed with PANTHER software. Regulatory network prediction was performed by Ingenuity Pathway Analysis (IPA) software (Qiagen Inc. https://digitalinsights.qiagen.com/products-overview/discovery-insights-portfolio/analysis-and-visualization/qiagen-ipa/) utilising Ingenuity Knowledge Base, a highly structured repository of biological interactions and functional annotations.

### Statistical analyses

The protein and phosphopeptide intensities within each condition were normalised to the total peptide amount. The fold changes of the different conditions were estimated using the average control abundances from five biological replicates. The log2 transformed protein ratios and differential expression were analysed using limma and stats packages [18]. Phosphopeptide abundances were further normalized against the abundance of the non-modified proteins. The ratios of proteins were regarded as being significantly changed between conditions compared if the q value was less than 0.05.

For comparing multiple groups, one-way ANOVA was performed followed by Tukey’s post-hoc test. Groups were considered to be significantly different when p value was less than 0.05.

Differential expression of IPA predicted regulatory genes among human multiple sclerosis lesion types vs. nonaffected white matter (NAWM) was identified by using the edgeR package (3.8) software [19] from the database created previously [20]. Adjusted p value filtering using the procedure of Benjamini and Hochberg was used to establish significant differences.

## Results

### De- and remyelination affected about 5.5% of the proteins identified

**Fig 1:**
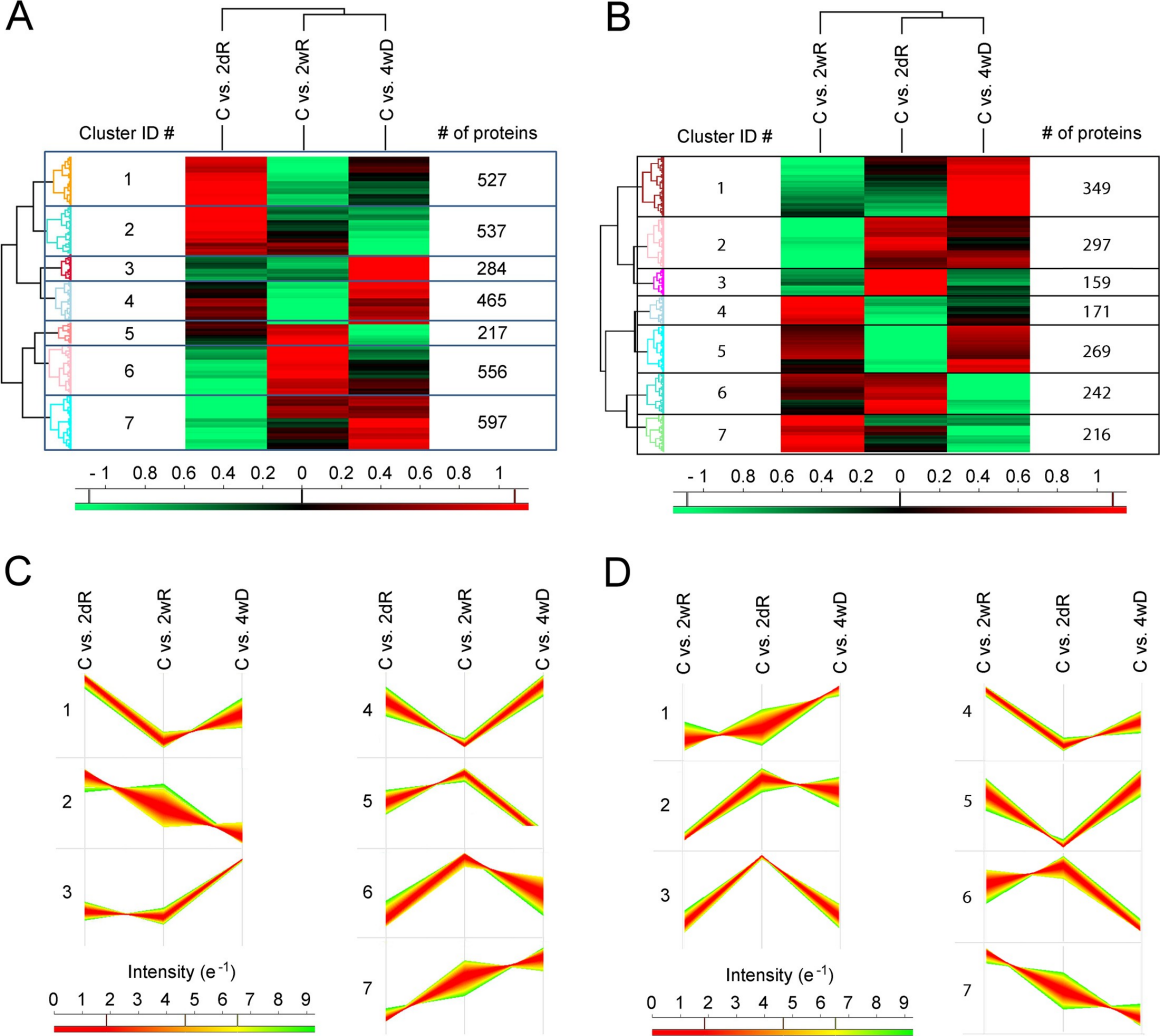
Cluster analysis. Mice were treated or not (C) with 0.2% cuprizone in their food for 4 weeks then were sacrificed after termination of the treatment (4wD), and 2 (2dR) or 14 (2wR) days later. Cluster analysis of 3183 unmodified **(A** and **C)** and 1703 single- or multi phosphorylated and/or gycosylated proteins **(B** and **D)** identified in corpus callosum homogenate of the mice were performed by using the Perseus software platform (http://www.perseus-framework.org). Heat maps **(A** and **B)** and pattern of protein concentration changes in the treatment groups **(C** and **D)** are presented. The concentrations were normalised to those of C group and were expressed in log_2_(fold change) values. Heat map scales are z score based, number of the proteins in a given cluster **(A** and **B)** as well as cluster ID next to a given cluster pattern **(C** and **D)** are indicated.

In an attempt to identify key elements regulating de- and remyelination, we isolated and homogenized the corpus callosum from mice exposed to CPZ, and performed liquid-chromatography mass-specrometry analysis from the homogenates. Following the same protocol we have used for studying transcriptome changes and the effect of microRNA-146a on CPZ-induced demyelination [14, 21], we induced demyelination in all mice except the controls (C) for 4 weeks with 0.2% CPZ (4wD), then allowed remyelination by terminating CPZ supplementation for 2 days (2dR) and 2 weeks (2wR). From the 20 samples (5 mice per group) we could identify altogether 3183 unmodified proteins. In addition to the unmodified ones, we detected 6017 single- or multi phosphorylated and/or gycosylated (PTM) peptides. Based on them, we identified 1703 PTM proteins. We performed clustering of these proteins for visualization how their level changed during de- and remyelination. Initially, pattern of protein level changes became markedly more coherent as the number of clusters was increased. However, the coherency did not increase significantly by increasing the number of clusters from 7 to 8. Therefore, we selected 7 clusters for both the unmodified (Fig 1A) and PTM proteins (Fig 1B). However, the proportional distribution (Fig 1A and 1C) of the proteins among the clusters differed. So did the directions of the protein level changes (Fig 1C and 1D) among the different experimental groups within the given clusters. Furthermore, while for the unmodified proteins, demyelination was found to be more closely related to late remyelination (Fig 1A) by the Perseus algorithm, it was early remyelination for the PTM proteins (Fig 1B). All these data indicated that clustering the whole protein population, although provided a basic visualisation of the changes the proteins followed during de- and remyelination, did not provide any clear lead toward finding proteins that conceivably regulate de- or remyelination.

**Fig 2:**
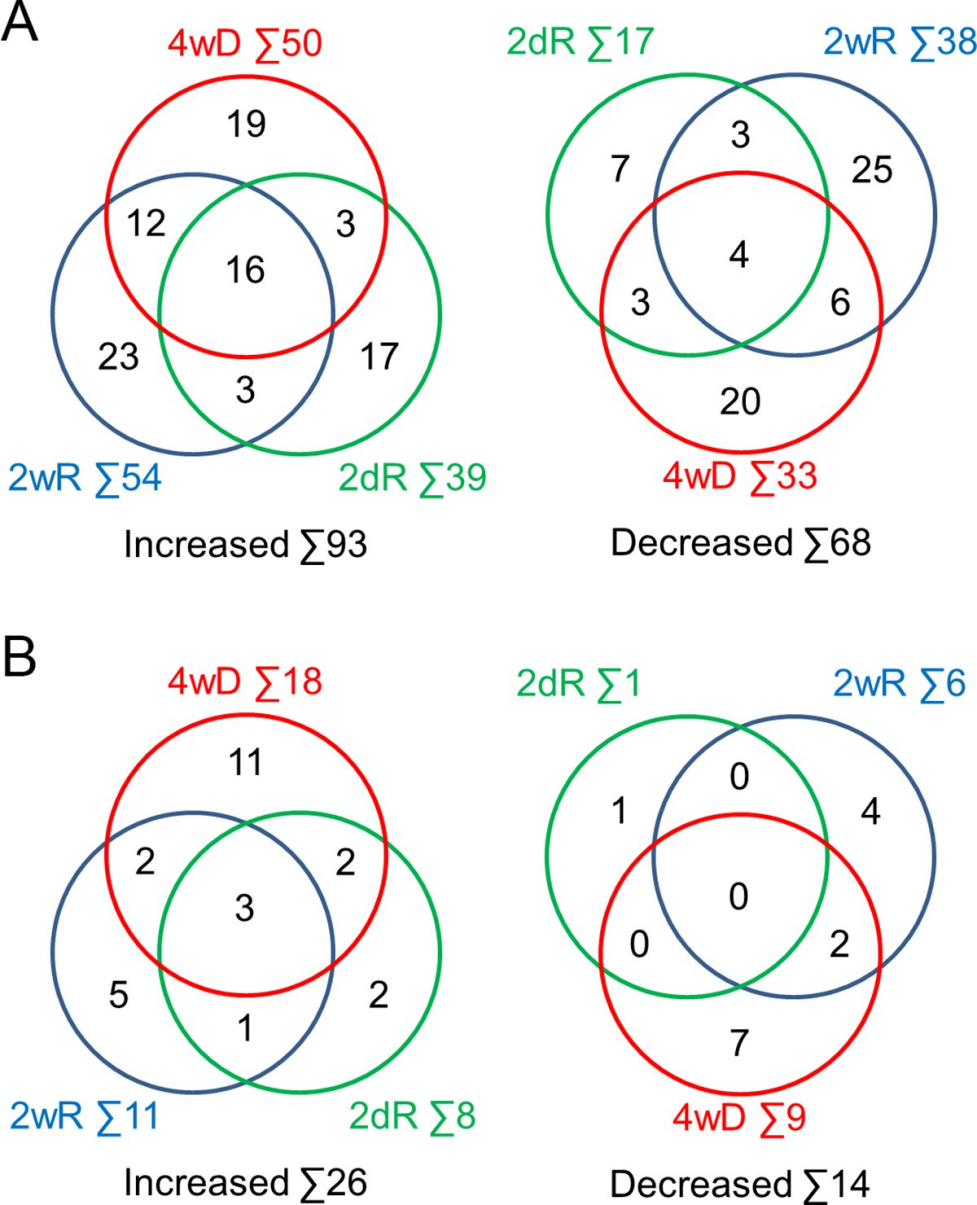
Venn diagrams for the proteins significantly altered in the experimental groups. Distribution of unmodified **(A)** and single- or multi phosphorylated and/or gycosylated proteins **(B)** among the different experimental groups are presented. Numbers indicate the number of the proteins in Tables 1 and 2.

For further analysis, we used those 1970 unmodified and 1255 PTM proteins only that were present in at least 80% of the samples. Furthermore, we considered the protein level to be significantly altered when (i) the protein’s identification was based on at least two unique peptides, (ii) it differed from the control significantly (q<0.05) and (iii) level of the unique peptides changed parallel to each-other. We identified 161 such unmodified proteins (Table 1); 93 of increased and 68 of decreased level during de- and remyelination, respectively (Fig 2A). Out of the 1255 single- or multi phosphorylated and/or gycosylated proteins, 40 were significantly affected by de- and remyelination (Table 2 and Fig 2B). Interestingly, these protein populations did not overlap; we could not find any protein that significantly changed one way by the CPZ treatment and the other way when CPZ treatment was discontinued. We found only nine proteins that were identified both as PTM and unmodified ones (Tables 1 and 2; grey shading). Among the PTM proteins of elevated level, 30% were phosphorylated (Table 2; yellow shading), the rest were N-glycosylated. In contrast, 92% of the PTM proteins of decreased level were at least monophosphorylated (Table 2; yellow shading).

**Table 1:** Concentrations of unmodified proteins significantly affected by de- and remyelination.

access #	Protein	4wD	q	2dR	Q	2wR	q
O35639	Annexin A3	1.129	0.000	0.877	0.004	0.613	0.027
O89017	Legumain	1.591	0.000	1.025	0.002	0.708	0.009
P03995	Glial fibrillary acidic protein	1.465	0.000	1.333	0.000	1.733	0.000
P07356	Annexin A2	0.947	0.001	0.741	0.016	0.567	0.017
P14106	Complement C1q subcomponent subunit B	1.126	0.000	0.792	0.007	1.578	0.000
P16110	Galectin-3	2.181	0.002	1.616	0.002	1.632	0.003
P18242	Cathepsin D	1.262	0.000	0.753	0.003	0.729	0.000
P20152	Vimentin	1.562	0.000	1.107	0.000	1.275	0.000
P24452	Macrophage-capping protein	1.196	0.000	0.815	0.017	1.457	0.000
P26041	Moesin	0.885	0.007	0.841	0.000	0.627	0.000
P31786	Acyl-CoA-binding protein	0.700	0.026	0.792	0.006	0.505	0.001
Q61233	Plastin-2	1.009	0.007	0.735	0.011	0.789	0.000
Q8BTM8	Filamin-A	0.701	0.002	0.434	0.031	0.276	0.049
Q99L04	Dehydrogenase/reductase SDR family 1	1.024	0.001	0.698	0.011	0.712	0.000
Q9DAW9	Calponin-3	0.949	0.002	0.570	0.027	0.752	0.000
Q9WUU7	Cathepsin Z	1.538	0.001	0.926	0.006	1.181	0.004
P20060	Beta-hexosaminidase subunit beta	1.168	0.000	0.503		0.895	0.000
P29758	Ornithine aminotransferase, mitochondrial	0.404	0.032	0.385		0.304	0.040
Q9JLF6	Gamma-glutamyltransferase K	1.605	0.006	1.204		1.714	0.004
O08709	Peroxiredoxin-6	0.598	0.014	0.448		0.759	0.000
P10605	Cathepsin B	0.749	0.003	0.137		0.511	0.004
P16045	Galectin-1	1.062	0.002	0.508		0.749	0.002
P68033	Actin, alpha cardiac muscle 1	1.886	0.002	0.986		1.404	0.004
P84075	Neuron-specific calcium-binding protein	0.775	0.012	0.390		0.806	0.000
P97371	Proteasome activator complex subunit 1	0.630	0.019	0.337		0.480	0.003
Q02105	Complement C1q subcomponent subunit C	1.102	0.001	0.504		1.725	0.000
Q61599	Rho GDP-dissociation inhibitor 2	0.805	0.001	0.420		0.387	0.026
Q8BMS1	Trifunctional enzyme subunit alpha	0.437	0.030	0.307		0.318	0.026
P48036	Annexin A5	0.349		0.464	0.030	0.396	0.004
P61205	ADP-ribosylation factor 3	1.228		0.920	0.043	0.779	0.004
P58771	Tropomyosin alpha-1 chain	0.231		0.671	0.039	1.172	0.004
P47955	60S acidic ribosomal protein P1	0.541		0.414		0.315	0.027
P56564	Excitatory amino acid transporter 1	0.390		0.232		0.451	0.012
P98086	Complement C1q subcomponent subunit A	0.820		0.596		1.510	0.000
Q9EQU5	Protein SET	0.423		0.160		0.477	0.004
Q06890	Clusterin	0.039		0.346		0.779	0.000
O35658	Complement component 1 Q-binding protein	0.431		0.265		0.536	0.047
P03921	NADH-ubiquinone oxidoreductase chain 5	0.469		0.528		0.393	0.029
P08226	Apolipoprotein E	0.518		0.397		0.846	0.000
P28798	Granulins	0.926		0.080		1.449	0.004
P29416	Beta-hexosaminidase subunit alpha	0.578		0.496		0.516	0.026
P42225	Signal transducer/transcription activator 1	0.482		0.512		0.732	0.034
P51880	Fatty acid-binding protein, brain	0.810		0.291		0.880	0.000
P55264	Adenosine kinase	0.013		-0.182		0.321	0.038
P56565	Protein S100-A1	0.582		0.614		0.664	0.001
P62082	40S ribosomal protein S7	0.335		0.190		0.271	0.048
P63040	Complexin-1	0.482		-0.019		0.656	0.003
P70202	Latexin	0.359		0.233		0.446	0.003
P97372	Proteasome activator complex subunit 2	0.634		-0.456		0.905	0.014
P99027	60S acidic ribosomal protein P2	0.357		0.244		0.544	0.006
Q61207	Sulfated glycoprotein 1	0.444		0.299		0.612	0.006
Q8BGC4	Zinc-binding ADH domain-containing protein 2	0.243		-0.274		0.479	0.032
Q9Z127	Large neutral amino acids transporter 1	0.519		0.310		0.689	0.004
Q9Z1T2	Thrombospondin-4	-0.661		0.211		1.371	0.016
P17225	Polypyrimidine tract-binding protein 1	0.681	0.009	0.473	0.021	0.221	
Q62417	Sorbin and SH3 domain-containing protein 1	0.530	0.016	0.447	0.034	0.212	
Q9JM63	ATP-sensitive inward rectifier K^+^ channel 10	0.730	0.024	0.940	0.004	0.222	
P62849	40S ribosomal protein S24	0.830	0.007	0.246		0.593	
Q00915	Retinol-binding protein 1	0.622	0.009	0.166		0.431	
Q8R0Y6	10-formyltetrahydrofolate dehydrogenase	0.331	0.038	0.164		0.270	
Q9D379	Epoxide hydrolase 1	0.904	0.002	0.396		0.430	
Q9DCN2	NADH-cytochrome b5 reductase 3	0.714	0.001	0.368		0.343	
Q9JJU8	SH3-binding glutamic acid-rich-like protein	0.480	0.036	0.311		0.280	
Q9WV32	Arp 2/3 complex subunit 1B	0.691	0.046	0.344		0.409	
O08677	Kininogen-1	0.818	0.042	0.302		-0.039	
O88958	Glucosamine-6-phosphate isomerase 1	0.614	0.040	0.314		0.374	
P11352	Glutathione peroxidase 1	0.500	0.019	0.260		-0.022	
P17047	Lysosome-associated glycoprotein 2	0.612	0.006	0.346		0.218	
P26039	Talin-1	0.646	0.012	0.396		0.244	
Q05816	Fatty acid-binding protein, epidermal	0.939	0.007	0.053		0.468	
Q3UHB1	5'-nucleotidase domain-containing protein 3	0.504	0.036	0.339		0.167	
Q62348	Translin	0.524	0.046	0.333		0.103	
Q8VDD5	Myosin-9	0.535	0.035	0.301		0.230	
Q9D0S9	Histidine triad nucleotide-binding protein 2	0.403	0.046	0.251		0.082	
Q9Z110	Delta-1-pyrroline-5-carboxylate synthase	0.550	0.020	0.313		0.013	
Q9Z1E4	Glycogen [starch] synthase	0.966	0.046	-0.055		0.331	
O54983	Ketimine reductase mu-crystallin	0.578		0.489	0.026	0.193	
Q08331	Calretinin	0.695		1.042	0.038	0.179	
Q9DB73	NADH-cytochrome b5 reductase 1	0.326		0.404	0.039	0.130	
Q9Z0F7	Gamma-synuclein	0.709		0.696	0.014	-0.224	
P03888	NADH-ubiquinone oxidoreductase chain 1	0.977		0.839	0.039	0.583	
P07309	Transthyretin	0.249		0.743	0.004	-0.047	
P07724	Serum albumin	0.364		0.572	0.019	0.167	
P10126	Elongation factor 1-alpha 1	0.102		0.555	0.011	-0.072	
P23492	Purine nucleoside phosphorylase	0.248		0.539	0.011	0.055	
P34884	Macrophage migration inhibitory factor	0.882		0.502	0.026	0.158	
P63038	60 kDa heat shock protein, mitochondrial	0.233		0.412	0.039	0.152	
Q6PE15	Mycophenolic acid acyl-glucuronide esterase	0.520		0.477	0.039	0.202	
Q9CQI6	Coactosin-like protein	0.129		0.554	0.039	0.228	
Q9D1I5	Methylmalonyl-CoA epimerase	0.121		0.695	0.039	0.050	
Q9DBS2	Tumor p63-regulated gene 1-like protein	0.548		0.544	0.030	0.172	
Q9QYG0	Protein NDRG2	0.731		0.595	0.038	0.380	
Q9R0P9	Ubiquitin hydrolase L1	0.399		0.624	0.011	0.151	
P97315	Cysteine and glycine-rich protein 1	-0.704	0.007	-0.667	0.004	-0.390	0.014
Q5EBJ4	Ermin	-1.053	0.000	-0.818	0.033	-0.641	0.020
Q8K298	Actin-binding protein anillin	-0.681	0.008	-0.912	0.011	-0.902	0.000
Q8R3P0	Aspartoacylase	-0.617	0.016	-0.883	0.000	-0.839	0.000
Q8BR63	Protein FAM177A1	-0.646	0.037	-0.698		-0.846	0.002
P16330	2',3'-cyclic-nucleotide 3'-phosphodiesterase	-0.492	0.042	-0.537		-0.593	0.004
P23927	Alpha-crystallin B chain	-0.836	0.011	-0.446		-0.727	0.029
Q05BC3	Echinoderm microtubule-associated 1	-0.707	0.016	-0.467		-0.422	0.012
Q9CRB6	Tubulin polymerization-promoting protein 3	-0.658	0.007	-0.388		-0.702	0.000
Q9D8B7	Junctional adhesion molecule C	-1.109	0.003	-0.577		-0.665	0.027
Q7M750	Opalin	-0.577		-0.803	0.007	-0.937	0.000
Q9D154	Leukocyte elastase inhibitor A	-0.442		-0.606	0.006	-0.596	0.003
Q5SYD0	Unconventional myosin-Id	-0.251		-0.505	0.016	-0.566	0.006
O54988	STE20-like serine/threonine-protein kinase	-0.290		-0.238		-0.271	0.032
Q8CAY6	Acetyl-CoA acetyltransferase, cytosolic	-0.771		0.074		-0.407	0.007
O70172	PI 5-phosphate 4-kinase type-2 alpha	-0.183		-0.352		-0.401	0.008
Q921C1	Gap junction gamma-3 protein	-2.460		0.200		-1.248	0.033
Q62433	Protein NDRG1	-0.305		-0.404		-0.490	0.005
P08553	Neurofilament medium polypeptide	-0.232		-0.158		-0.387	0.029
P84096	Rho-related GTP-binding protein RhoG	-0.434		-0.454		-0.635	0.001
P40237	CD82 antigen	-0.509		-0.475		-0.707	0.033
P40240	CD9 antigen	-0.232		-0.353		-0.732	0.000
P00920	Carbonic anhydrase 2	-0.469		-0.417		-0.599	0.006
P61329	Fibroblast growth factor 12	-0.662		0.073		-0.599	0.000
P62746	Rho-related GTP-binding protein RhoB	-0.422		-0.190		-0.337	0.014
P97370	Na^+^/K^+^-transporting ATPase beta-3	-0.430		-0.383		-0.289	0.037
Q3TUF7	YEATS domain-containing protein 2	-0.408		-0.622		-0.934	0.037
Q64012	RNA-binding protein Raly	-0.353		0.021		-0.883	0.004
Q8BGN3	Ectonucleotide phosphodiesterase 6	-0.296		-0.328		-0.588	0.006
Q8BVI4	Dihydropteridine reductase	-0.197		-0.163		-0.315	0.027
Q8CIG8	Protein arginine N-methyltransferase 5	-0.079		0.078		-0.321	0.043
Q8R366	Immunoglobulin superfamily member 8	-0.132		-0.163		-0.345	0.023
Q64487	Receptor-type tyrosine-phosphatase delta	-0.380		-0.094		-0.295	0.034
Q8VDQ8	Sirtuin-2	-0.154		-0.097		-0.501	0.006
Q99J77	Sialic acid synthase GN = Nans	-0.644		0.072		-0.366	0.032
Q9EQF6	Dihydropyrimidinase-related protein 5	-0.072		-0.001		-0.621	0.006
Q9R1V7	Disintegrin and MPD-containing protein 23	-0.219		-0.078		-0.313	0.044
Q8BH66	Atlastin-1	-0.349		-0.107		-0.259	0.038
Q920E5	Farnesyl pyrophosphate synthase	-0.502	0.019	-0.452	0.039	-0.244	
Q9WV27	Na^+^/K^+^-transporting ATPase alpha-4	-0.703	0.036	-0.916	0.011	-0.415	
Q9Z2Y3	Homer protein homolog 1	-0.462	0.046	-0.366	0.039	-0.090	
P00158	Cytochrome b	-1.776	0.003	-0.544		-0.483	
P62071	Ras-related protein R-Ras2	-0.868	0.028	-0.314		-0.422	
Q8C078	Calcium/calmodulin-dependent PKK 2	-0.851	0.002	-0.223		-0.337	
P07759	Serine protease inhibitor A3K	-0.636	0.042	-0.504		-0.052	
P11881	Inositol 1,4,5-trisphosphate receptor type 1	-0.396	0.046	-0.153		-0.084	
P12023	Amyloid beta A4 protein	-0.593	0.005	-0.315		-0.179	
P15105	Glutamine synthetase	-0.574	0.006	-0.240		-0.162	
P28661	Septin-4	-0.353	0.046	-0.493		-0.305	
Q14BB9	MAP6 domain-containing protein 1	-0.586	0.036	-0.357		-0.167	
Q5SVL6	Rap1 GTPase-activating protein 2	-0.498	0.019	0.040		-0.186	
Q8CHH9	Septin-8	-0.348	0.046	-0.387		-0.221	
Q61699	Heat shock protein 105 kDa	-0.321	0.032	-0.137		-0.080	
Q6PDY2	2-aminoethanethiol dioxygenase	-0.505	0.041	-0.387		-0.182	
Q80YN3	Breast carcinoma-amplified seq1 homolog	-0.860	0.000	-0.685		-0.552	
Q99PJ0	Neurotrimin	-0.453	0.020	-0.351		-0.145	
Q9EPL2	Calsyntenin-1	-0.564	0.011	-0.598		-0.083	
Q9QUR8	Semaphorin-7A	-0.425	0.032	-0.287		-0.206	
Q9WV34	MAGUK p55 subfamily member 2	-0.330	0.047	-0.339		-0.092	
P13020	Gelsolin	-0.425	0.028	-0.386		-0.005	
Q8CC35	Synaptopodin	-0.415	0.042	-0.488		-0.055	
Q3UTJ2	Sorbin and SH3 domain-containing protein 2	-0.271		-0.422	0.034	-0.069	
Q922U2	Keratin, type II cytoskeletal 5	-1.424		-1.565	0.050	-0.674	
P02535	Keratin, type I cytoskeletal 10	-0.873		-1.023	0.033	-0.561	
P35803	Neuronal membrane glycoprotein M6-b	-0.246		-0.413	0.043	-0.025	
Q8K2K6	Arf-GAP domain and FG repeat-containing 1	-0.295		-0.438	0.039	-0.040	
Q8K406	Leucine-rich repeat LGI family member 3	-0.207		-0.424	0.038	-0.255	
O35405	Phospholipase D3	-0.492		-0.637	0.002	-0.151	

The concentrations of the proteins in the different experimental groups were normalised to those of C group and were expressed in log_2_(fold change) values. Significant difference from the control is indicated by presenting the q value, and shading red the increases and green the decreases. Those proteins that were identified as both unmodified and single- or multi phosphorylated and/or gycosylated are shaded grey.

**Table 2:** Concentrations of single- or multi phosphorylated and/or gycosylated (PTM) proteins significantly affected by de- and remyelination.

Peptide sequence	access #	protein	4wD	q	2dR	q	2wR	q
AVLVNdNITTGEK	Q91XA2	Golgi membr. p1	1.961	0.001	1.339	0.011	1.225	0.030
VFIVPVGdNHSNIPFSR	Q8BG07	Phospholipase D4	1.319	0.047	1.679	0.003	1.861	0.008
APIPTALDTdNSSK	Q07797	Galectin-3-binding	1.994	0.001	2.113	0.000	1.993	0.001
ALGYEdNATQALGR			2.109	0.001	1.984	0.000	2.046	0.003
GLdNLTEDTYKPR			2.690	0.001	2.524	0.004	2.724	0.000
dNLTTLGIFGAATNK	Q80WV3	Carbohydrate ST2	1.601	0.003	0.729		1.305	0.035
LdNFTGPGEPDSLR	P11835	Integrin beta-2	1.384	0.011	0.954		1.606	0.006
VLTNQESPYQdNHTGR	P98086	Complement C1q	1.751		1.789	0.010	2.306	0.001
ATVdNDSGEYR	P08508	IG-G Fc receptor3	2.005		0.816		1.818	0.016
LLdNLTSPEATAK	O55026	EcTP DPase2	0.847		0.404		1.029	0.030
TGEPDEEEGTFRpSSIR	Q9Z239	Phospholemman	0.653		0.607		1.408	0.004
SPPDQSAVPNpTPPSTPVKLEEDLPQEPTSR	Q9QYC0	Alpha-adducin	0.387		-0.489		1.455	0.030
QELdNDSLQVAER	Q06890	Clusterin	-0.284		0.052		1.655	0.014
dNSTGCLK			0.558		0.895		1.896	0.014
ETISAIDpTSPK	Q3UH99	Protein shisa-6	1.839	0.015	1.513	0.014	1.288	
VLGFKPKPPKdNESLETYPLMMK	P14094	NA^+^/K^+^ ATPase	2.710	0.007	2.268	0.007	1.588	
dNSTFGSVEVFSLDPNK	P56528	cADP hydrolase 1	1.695	0.040	1.569		0.711	
YYHGELSYLdNVTRK	P18242	Cathepsin D	1.540	0.046	1.021		-0.217	
TPALpSPQRPLTTQQPQSGTLK	Q64332	Synapsin-2	1.376	0.011	0.438		0.418	
AWGISVLNPdNK	P31996	Macrosialin	2.719	0.003	1.881		1.794	
EAFdNETNQAIQTISR	P41233	ATP-BC A 1	1.022	0.040	0.720		0.874	
DdNATQEEILHYLEK	Q61207	Sulf. Glycoprot.1	1.476	0.014	1.156		1.357	
EGEEPTVYpSDDEEPK	O55022	Progesterone R	2.235	0.011	1.144		0.048	
VAAGHELQPLAIVDQRPSpSR	P23242	Gap junction α-1	1.228	0.041	0.961		0.490	
DLGPALAdNSSHDVK	P17439	Ceramidase	1.560	0.049	0.999		0.780	
pSAEDLTDGSYDDILNAEQLK	Q9D0L7	AR-containing p10	1.147	0.049	1.093		0.881	
EdNITAEALDLSLK	Q61704	Trypsin inhib.H3	3.325	0.002	1.380		0.900	
VVLHPdNHSVVDIGLIK	Q61646	Haptoglobin	2.244		3.230	0.014	0.405	
NLFLdNHSETASAK			0.920		3.621	0.012	0.725	
EEAKpSPGEAKpSPGEAK	P19246	Neurofilament H	2.150		2.889	0.048	-0.311	
GVVTNGLDVpSPAEEKKGEDSSDDK	P08553	Neurofilament M	-6.053	0.039	-1.546		-3.232	0.030
TTHYGSLPQKpSQHGR	P04370	Myelin basic prot.	-4.866		-1.751		-4.179	0.012
FFpSGDRGAPK			-0.702		-1.974		-3.758	0.014
NIVTPRpTPPPSQGK			-2.850	0.000	-1.920		-0.938	
NIVpTPRTPPPSQGK			-2.498	0.000	-1.810		-0.900	
EAENTANQAGNEpSPVQELRQDVSK	Q6NVE8	WDR-contain. p44	-1.297		-0.542		-2.456	0.014
SHTSEDARLdNIpTPNSGATGNNAGPK	Q62433	Protein NDRG1	-1.374		-0.832		-2.134	0.014
DLHESSFpSLSGSQIDDHVPK	Q9EQF6	Dhpyrimidinase 5	0.115		-0.181		-1.617	0.004
GKPVPIHGpSR	P16330	cNMP 3'-PDE	-1.541		-0.158		-1.568	0.013
HQPAApSPVVVR	Q9D2P8	Myelin-assoc. OBP	-2.272	0.001	-1.340		-0.945	
EHANIDAQSGSQAPNPSTpTISPGKSPPPAK	Q8VDQ8	Sirtuin-2	-1.130	0.008	-1.264		-0.653	
EHANIDAQSGSQAPNPpSTTISPGKSPPPAK			-1.406	0.012	-1.346		-0.586	
QPGFPQPSPSDDPSLpSPRQDR	Q91VC7	Phosphatase 1	-1.673	0.003	-1.635		-1.012	
pTPSPPEPEPAGTAQK	Q80YN3	BCAS 1 homolog	-1.072	0.019	-1.225		-0.539	
AVSpSPTVSR	Q7TQD2	Tubulin PPP	-2.207	0.027	-1.196		-0.459	
VVVHKETEIpTPEDGED	Q9WV92	Band 4.1-like p3	-4.207	0.003	-0.775		-0.200	
QKFHDpSEGDDTEETEDYR	Q8K019	Bcl-2-assoc. TF1	-2.924	0.041	-0.916		0.167	
LIDLEpSPTPESQK	Q8C0T5	SIPA 1-like p1	-0.581		-1.252	0.048	-0.689	

The concentrations of the proteins in the different experimental groups were normalised to those of C group and were expressed in log_2_(fold change) values. Peptide sequences are indicated, phosphorylated peptides are shaded yellow. Blank lines below a protein’s access # and name indicate that the particular protein was identified by more than one PTM peptides. Significant difference from the control is indicated by presenting the q value, and shading red the increases and green the decreases. Those proteins that were identified as both unmodified and single- or multi phosphorylated and/or gycosylated are shaded grey.

As we [15] and others [13, 22] demonstrated previously, 4 weeks of cuprizone treatment caused massive demyelination in the corpus callosum that was followed by rapid remyelination when the treatment was discontinued [13, 22]. Accordingly, we expected proteins specific for demyelination-, early- and late remyelination, i.e. proteins with levels that differed from the C group respectively in the 4wD, 2dR and 2wR groups only. Based on this definition, we identified 39 demyelination specific, 24 early- and 48 late remyelination specific unmodified (Fig 2A), and 18, 3 and 9 PTM proteins (Fig 2B), respectively. On the other hand, although in different extent, 30 unmodified and 6 PTM proteins were affected in two, while 20 unmodified and 3 PTM proteins in all three groups (Fig 2).

### Immunohistochemical analysis of phosphorylation during de- and remyelination

**Fig 3:**
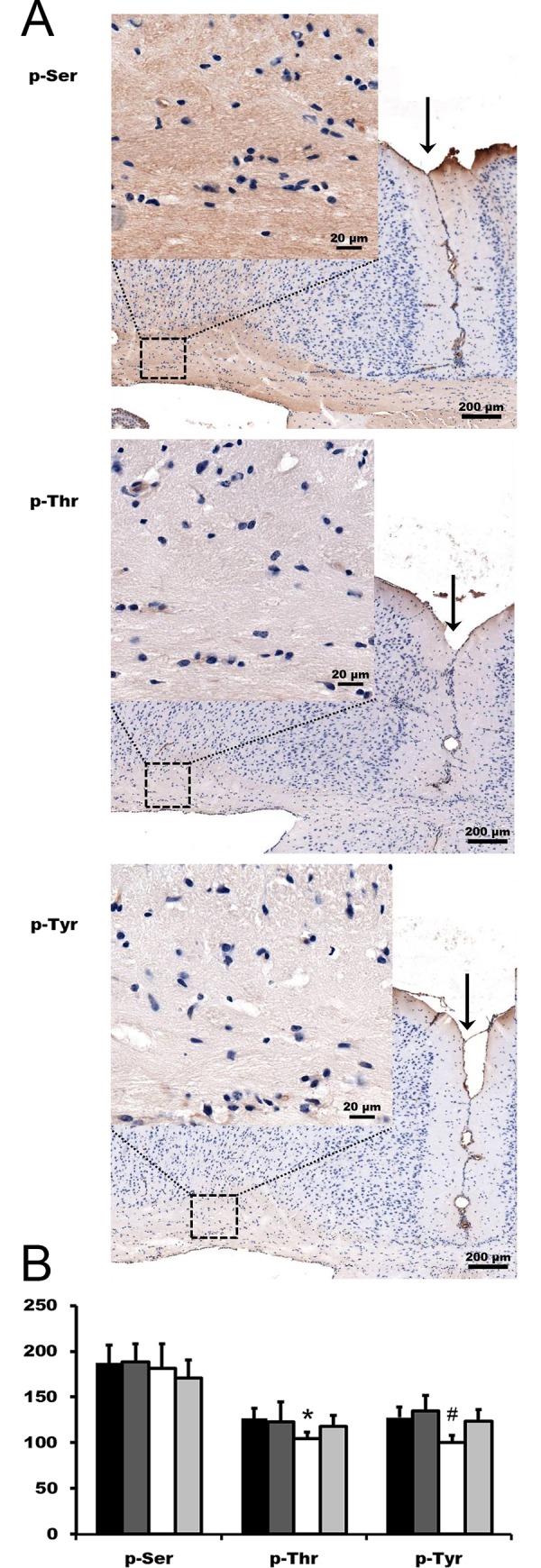
Immunohistochemical analysis of phosphorylation during de- and remyelination. We performed immunohistochemistry utilising phospho (p)-Ser, p-Thr and p-Tyr specific primary antibodies on brain sections of the animals (n = 3) from all four treatment groups. Representative sections are presented (**A**). For quantitative assessment of phosphorylation during de- and remyelination (**B**), all nuclei and staining artefacts were eliminated from the sections, and staining intensities of corpus callosum were normalised by an expert blind to the experiment to background i.e. left and right retrosplenial area, ventral part, layer 1 (lightly stained area containing few nuclei left and right to the arrow in **A**) by using Molecular Devices’ MetaXpress® image analyser software. Data are presented mean ± standard deviation % of the background (n = 3). Filled, dark grey, open and light grey bars denote control, 4-week demyelination, 2-day and 2-week remyelination groups, respectively. * significantly different from C; # significantly different from 4W.

Many cellular functions are regulated by phosphorylation, and the observed uneven distribution of phosphorylation among up- and downregulated PTM proteins raised the possibility of a specific role for phosphorylation during de- and remyelination. Accordingly, we performed immunohistochemistry utilising p-Ser, p-Thr and p-Tyr specific primary antibodies on brain sections of the animals from all four experimental groups. We observed intermediate to weak specific staining that was localised to the nerve fibres rather than the cell bodies or nuclei (Fig 3A). Seldom, staining of a nucleus or a cell body was found, however, no specific pattern or staining characteristric for a certain cell type could be identified (Fig 3A). In an attempt to correlate phosphorylation with either de- or remyelination, we measured staining intensities among the brain sections by using an image analyser software. We found up to about 9, 17 and 26% differences among the groups for Ser, Thr and Tyr phosphorylation, respectively (Fig 3B). Also, the staining intensities were lower in early remyelination than in the control and the demyelination groups for Thr and Tyr phosphorylation, respectively (Fig 3B).

### GO analysis of proteins affected by de- and remyelination

In order to identify specific biological processes, we performed over-representation test on the proteins affected by de- and remyelination. This test determines representation of the proteins in question in molecular function, biological process or cellular component categories selected as significant ones by the software over estimated representation of the mouse proteome in the same categories. We pooled the differentially regulated 192 PTM and unmodified proteins, and performed the test for biological processes. All the proteins but two were classified into 76 categories, an extract of which is presented in Table 3. The biological process categories included glial and neuronal function, metabolism, cell death, inflammatory response, protein and cation homeostasis, and cytoskeleton related processes (Table 3). Besides the expected oligodendrocyte compatible ones, the overrepresentation test indicated involvement of astrocyte and microglia related events such as glutamate metabolic process [23] and inflammatory response [24, 25]. These data are consistent with the accepted view that CPZ induced oligodendrocyte loss is accompanied by expansion and activation of microglia and astrocytes [26].

**Table 3:** Extract of panther biological process over-representation test for the proteins significantly altered in the experimental groups.

	# Mus	Client Text Box Input			
Biological process	musculus	#	expected	Fold Enrich.	q
myelin maintenance	15	3	0.13	23.47	2.73E-02
positive regulation of cholesterol transport	24	4	0.2	19.56	8.12E-03
negative regulation of catalytic activity	671	16	5.72	2.8	1.80E-02
neurofilament bundle assembly	3	2	0.03	78.23	3.82E-02
negative regulation of amyloid fibril formation	3	2	0.03	78.23	3.80E-02
regulation of postsynaptic membrane organization	21	4	0.18	22.35	5.78E-03
response to calcium ion	128	7	1.09	6.42	1.22E-02
synapse organization	258	11	2.2	5	2.69E-03
walking behavior	47	4	0.4	9.99	4.64E-02
response to toxic substance	287	9	2.45	3.68	4.90E-02
positive regulation of apoptotic process	615	14	5.24	2.67	4.88E-02
negative regulation of cell death	1018	22	8.68	2.54	7.08E-03
inflammatory response	429	12	3.66	3.28	2.55E-02
negative regulation of protein homooligomerization	11	4	0.09	42.67	1.18E-03
positive regulation of sodium ion export across plasma membrane	4	3	0.03	88.01	2.76E-03
cellular potassium ion homeostasis	14	3	0.12	25.15	2.45E-02
positive regulation of calcium ion transport	133	7	1.13	6.18	1.44E-02
actin filament organization	223	12	1.9	6.31	2.43E-04
negative regulation of blood coagulation	45	5	0.38	13.04	6.58E-03
response to caloric restriction	3	2	0.03	78.23	3.79E-02
Unclassified	1873	2	15.96	0.13	2.48E-03

Protein list from Tables 1 and 2 were combined and biological process over-representation test was performed by using Protein Analysis Through Evolutionary Relationships (PANTHER) classification system software (http://www.pantherdb.org). # Mus musculus and # denotes the number of proteins in the given category based on the mouse genome and the actual number of proteins in the aforementioned list, respectively. “Expected” indicates the expected number of proteins in the aforementioned list in the case of no over- or under-representation. Fold Enrich. = #/ expected; the fold over- or under-representation. Significance of the analysis is indicated by presenting the q value.

**Fig 4:**
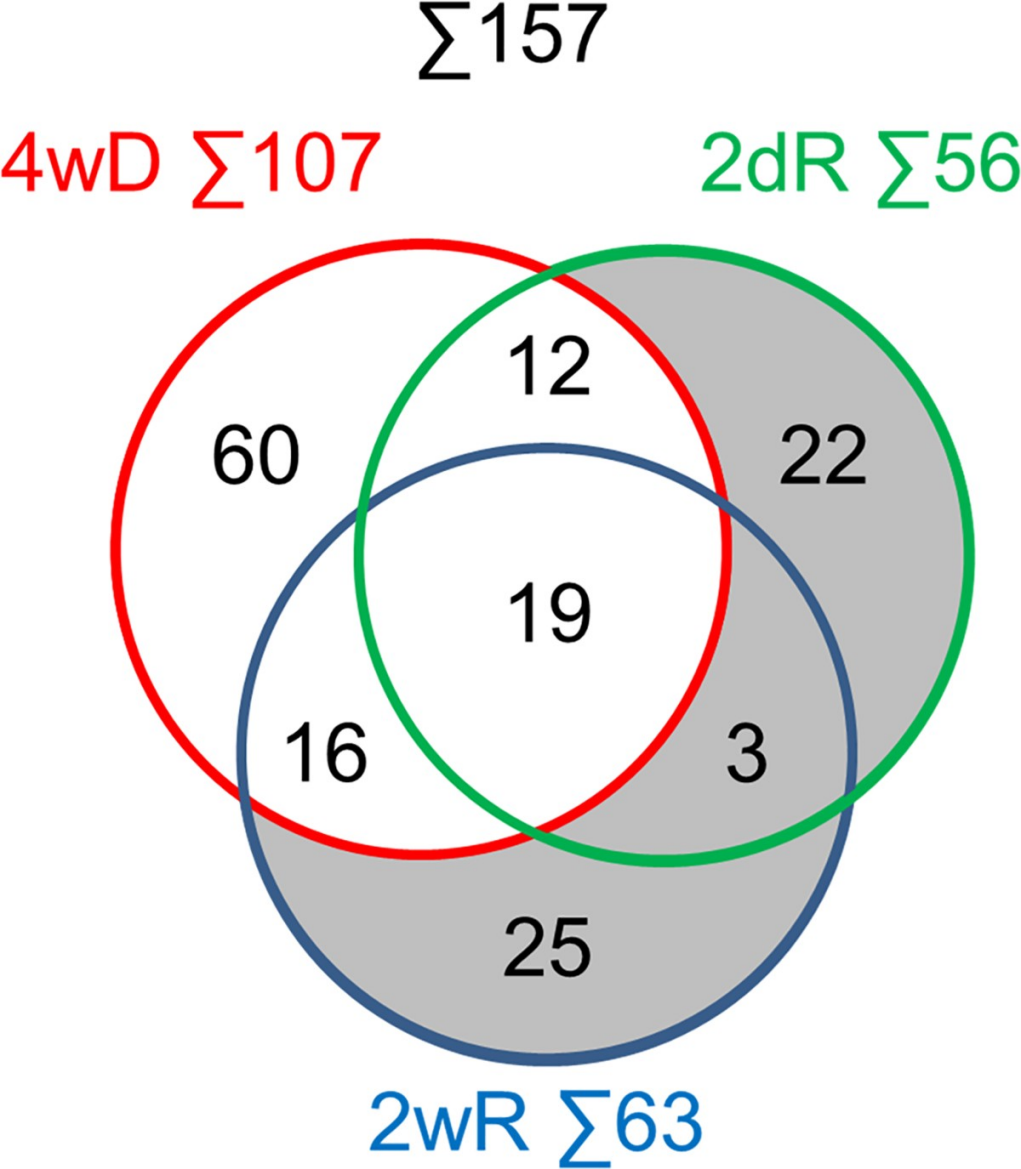
Venn diagrams for the shortlisted proteins. Those proteins of Tables 1 and 2 that were PANTHER unclassified or belonged to a biological process of smaller than 50% over- or under-representation were deleted from the list. Distribution of the 157 remaining unmodified and single- or multi phosphorylated and/or gycosylated proteins among the different experimental groups (see Fig 1) are presented. Remyelination only proteins are shaded.

In addition to the two unclassified proteins, we eliminated those that were involved in biological processes of less than 50% over- or under-representation. We divided the remaining 157 proteins (Fig 4) into remyelination only (RO, Fig 4 shaded) and demyelination related (DR, Fig 4 unshaded) groups and performed over-representation test on them. The biological process over-representation test allocated the DR proteins into 9 categories (Table 4). For the RO proteins, the single significant category was nervous system development that included myelination as the most specific subcategory (Table 5). Interestingly, a biological process over-representation test on the 60 demyelination specific proteins of the DR group did not identify any significant process.

**Table 4:** Panther biological process over-representation test for the shortlisted demyelination related proteins.

	# Mus	Client Text Box Input			
Biological process	musculus	#	expected	Fold Enrich.	q
regulation of synaptic plasticity	207	8	0.97	8.29	1.16E-02
regulation of trans-synaptic signaling	506	11	2.36	4.66	2.99E-02
regulation of biological process	11120	72	51.87	1.39	4.65E-02
biological regulation	11734	77	54.73	1.41	1.59E-02
regulation of biological quality	3830	43	17.87	2.41	1.45E-04
actin filament organization	223	8	1.04	7.69	1.62E-02
supramolecular fiber organization	444	14	2.07	6.76	2.13E-04
actin cytoskeleton organization	462	12	2.16	5.57	4.65E-03
cytoskeleton organization	1004	20	4.68	4.27	2.28E-04
actin filament-based process	520	13	2.43	5.36	3.00E-03
cellular metal ion homeostasis	551	11	2.57	4.28	3.97E-02
metal ion homeostasis	629	12	2.93	4.09	3.18E-02
cation homeostasis	696	13	3.25	4	2.77E-02
ion homeostasis	777	13	3.62	3.59	4.52E-02
homeostatic process	1588	20	7.41	2.7	3.13E-02
inorganic ion homeostasis	711	13	3.32	3.92	3.02E-02
cellular cation homeostasis	610	12	2.85	4.22	2.93E-02
cellular ion homeostasis	624	12	2.91	4.12	3.09E-02
cellular homeostasis	828	14	3.86	3.62	3.02E-02
regulation of cellular component biogenesis	908	16	4.24	3.78	1.06E-02
regulation of cell migration	870	14	4.06	3.45	4.00E-02
regulation of localization	2764	33	12.89	2.56	6.69E-04
regulation of transport	1897	24	8.85	2.71	1.03E-02
regulation of cellular component organization	2479	31	11.56	2.68	8.18E-04
system development	4126	37	19.25	1.92	3.13E-02
positive regulation of biological process	5897	48	27.51	1.75	2.09E-02

Those proteins of Tables 1 and 2 that were PANTHER unclassified or belonged to a biological process of smaller than 50% over- or under-representation were deleted from the list. Demyelination related proteins were classified as those 107 unmodified and single- or multi phosphorylated and/or gycosylated proteins that were significantly different from the control in the cuprizone treated group (Fig 4, unshaded). For the explanation, see Table 3.

**Table 5:** Panther biological process over-representation test for the shortlisted remyelination only proteins.

	# Mus	Client Text Box Input			
Biological process	musculus	#	expected	Fold Enrich.	q
myelination	103	6	0.23	25.98	2.45E-03
axon ensheathment	105	6	0.24	25.48	9.08E-04
ensheathment of neurons	105	6	0.24	25.48	1.36E-03
nervous system development	2178	18	4.88	3.69	2.20E-03

Those proteins of Tables 1 and 2 that were PANTHER unclassified or belonged to a biological process of smaller than 50% over- or under-representation were omitted. Remyelination only proteins were classified as those 50 unmodified and single- or multi phosphorylated and/or gycosylated proteins that were significantly different from the control in the early and late remyelination groups only (Fig 4, shaded). For the explanation, see Table 3.

**Fig 5:**
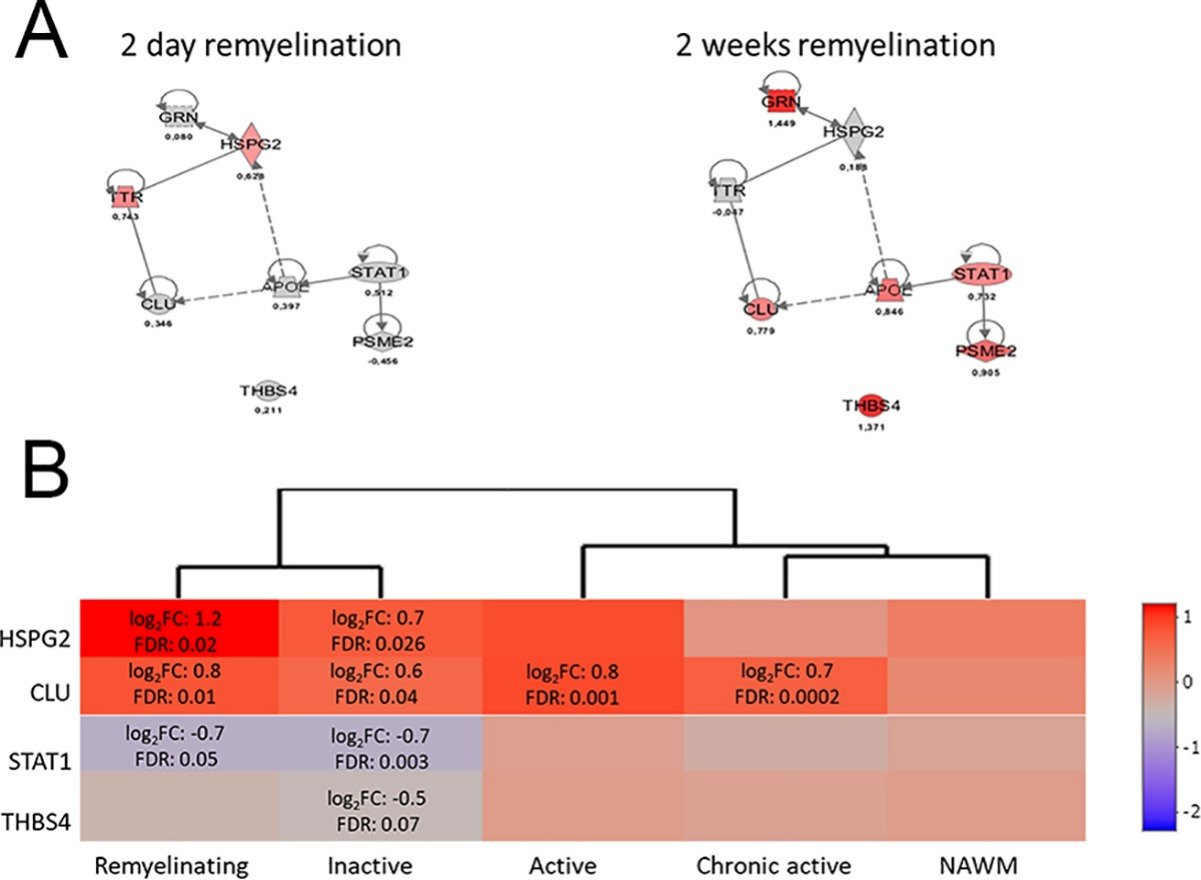
IPA regulator network analysis of RO proteins. IPA regulator network analysis was performed on the 2dR and 2wR groups of RO proteins. The resulting network is presented (**A**). Occurrence of the network orthologues among genes expressed differentially between human multiple sclerosis lesion types vs. NAWM [20] was studied by using the edgeR package (3.8) software [19]. The results are presented as a heat map. Key for molecule shapes and colors as well as relationship labels is published on http://qiagen.force.com/KnowledgeBase/articles/Basic_Technical_Q_A/Legend. Significant changes represented by node coloring based on the fold changes in protein levels.

To further study the RO proteins (Fig 4, shaded) for their involvement in mechanisms regulating remyelination, we used Ingenuity Pathway Knowledge Base to select a group of interconnected nodes to assess their cellular level changes during the phases of 2dR and 2wR. The analyses performed on the two remyelination groups resulted the same sole network of eight members (Fig 5A). In early remyelination, two of them were upregulated and the other six members not affected significantly, while the reverse pattern was observed during late remyelination (Fig 5A). We searched occurrence of the eight genes among genes expressed differentially between human multiple sclerosis lesion types vs. NAWM [20]. As we found, four of them were differentially expressed in at least one of the lesion types, and perlecan (*HSPG2*) gene was the most upregulated in remyelinating lesions (Fig 5B). In remyelinating multiple sclerosis lesions, the gene expression pattern of the identified experimental remyelination network orthologues was more consistent with early remyelination in the CPZ model, i.e. upregulation of *HSPG2* and downregulation of signal transducer and activator of transcription 1 (*STAT1*) and Thrombospondin-4 (*THBS4*) (Fig 5B).

## Discussion

Due to the lack of clear concept for the aetiology of multiple sclerosis, clinical treatment strategies are limited to delaying progression of the disease by suppressing inflammation [27]. Recently, proteomic analysis of CPZ treated mice’s brain samples was suggested as a potentially useful approach for finding therapeutic targets enabling causal treatment of the disease [28]. However, none of the previous studies [10–12] targeted the corpus callosum, where CPZ-induces the most pronounced demyelination [13]. Therefore, we assessed proteomic changes during de- and remyelination at different time points in the isolated corpus callosum of CPZ treated mice.

Although all previous proteomic studies used mice of the C57BL/6 genetic background, there were differences between genders of the animals, age of the mice at the start and length of CPZ treatment, and the timing of sample acquisition during de- and remyelination [10–12]. As we demonstrated previously [15], CPZ induced demyelination increases in extent rapidly during the first 4 weeks, then at a much slower rate for the following 2 weeks before the time window for regeneration closes [13]. Accordingly, we followed the same protocol as previously [14, 21], namely, 4 weeks of CPZ treatment (demyelination) followed by 2 (early remyelination) and 14 (late remyelination) days of recovery after termination of the CPZ treatment. Proteomic analysis of the corpus callosums resulted in altogether 4886 proteins, however, initial clustering of them did not indicate any group whose concentration changes followed a pattern indicative of de- and remyelination regulators (Fig 1).

We could select altogether 192 proteins whose concentration was significantly different from the control in at least one experimental group (Tables 1 and 2). They represented a high variety of molecular functions, few of which were characteristic of de- and remyelination processes. Out of the altogether 57 demyelination specific proteins, 25 had more than 1.5 fold of increased and 14 of decreased concentration (Tables 1 and 2). Among the latter, myelin basic protein (MBP) decreased to 13.9 while myelin-associated oligodendrocyte basic protein to 20.8% of their respective level in the control group (Table 2) clearly in accord with the massive demyelination and oligodendrocyte loss occurring in the 4wD group [14]. We identified myelin basic protein based on three of its phosphopeptides; one of them bearing two threonine phosphorylation sites (Table 2). Phosphorylation of these sites by mitogen activated protein kinases (MAPKs) was reported to dramatically reduce the protein’s binding to negatively charged lipid bilayers [29]. Considering that MAPKs are activated during the treatment [15], these phosphorylation changes are consistent with the cuprizone induced demyelination. Additionally, besides 17 proteins involved in lipid metabolism, we found two more myelin-associated proteins; 2',3'-cyclic-nucleotide 3'-phosphodiesterase [30] and ermin [31]. Interestingly, the latter occurred at decreased concentrations in all three experimental conditions (Table 1) that is hard to harmonize with its role in cytoskeletal rearrangements during myelinogenesis and maintenance of myelin sheath’s stability [31]. In line with the expectations, majority of the 27 early and 54 late remyelination specific proteins (Tables 1 and 2) were associated with myelin sheath, neuronal processes, synapse and cytoskeleton organisation (Table 5). However, out of the major oligodendrocyte marker proteins we found PTM form of myelin-associated oligodendrocyte basic protein and MBP only. On the other hand, we found decreased phosphorylated MBP levels (Table 2) as others did previously in multiple sclerosis patients’ and cuprizone mice’s brains [32]. Furthermore, our finding that all but one decreased PTM proteins were phosphorylated is in line with the view that decreased phosphorylation could be part of demyelination pathogenesis [32]. Unfortunately, the immunohistochemical study we performed to assess protein phosphorylation during de- and remyelination proved to be inconclusive. Although we found that Ser, Thr and Tyr phosphorylation was localised to the white matter, it seemed to be associated with the nerve fibres, did not change considerably among the groups, and no clear pattern or cell type specific staining could be identified (Fig 3).

In contrast to the remyelination only proteins, demyelination-related proteins were not associated (Table 4) with their respective expected biological process categories such as oligodendrocyte apoptosis, demyelination, oxidative stress or mitochondrial damage. Instead, a number of DR proteins were categorized into biological processes related to cytoskeletal and organelle reorganization, metal ion homeostasis and migration; processes most probably associated with astrocytosis and microglia activation [26]. Furthermore, the 60 demyelination only DR proteins could not be classified into any biological process category indicating that classification of these proteins with acceptable statistical significance was possible only when they were combined with the proteins that had altered level in two or all three experimental groups.

IPA network construction performed on early and late RO proteins resulted in a sole network, the members of which were activated in an inverse pattern during early vs. late remyelination (Fig 5A). Four out of the eight members were differentially expressed between human multiple sclerosis lesion types vs. NAWM (Fig 5B). Clusterin (*CLU*) that we found to be upregulated in all lesion types functions as an extracellular chaperone. It prevents aggregation of non-native proteins and maintains them in a state appropriate for refolding by ATPase chaperones [33]. In agreement with our results, *CLU* mRNA levels were reported to be elevated in glial fibrillary acidic protein-positive astrocytes in white matter lesions over NAWM, but not in grey matter of multiple sclerosis patients [34]. *HSPG2* that we found to be upregulated in inactive and remyelinating multiple sclerosis lesions is one of the largest extracellular matrix molecules. It was not so far associated with multiple sclerosis, however was suggested to be essential in establishing and patrolling tissue borders [35]. *STAT1* acts as a transcription factor for various growth factors. In agreement with other studies [36], we found it to be downregulated in inactive and remyelinating lesions. *THBS4* that we found to be downregulated in inactive multiple sclerosis lesions is an adhesive glycoprotein involved in cell-to-cell and cell-to-matrix interactions. Although not in association with multiple sclerosis, it was reported to play a role in tissue remodeling [37]. Taken together all information, a role for this predicted network in regulating remyelination processes does not seem compelling, therefore more studies are needed to identify mechanisms regulating de- and remyelination.

## Conclusions

Taken together all aforementioned data, proteomic analysis of the cuprizone treated corpus callosum seems more informative for the processes of remyelination over those of demyelination. In a broader sense, these results may indicate limitations of the cuprizone model in answering demyelination related specific questions in multiple sclerosis research by proteomics.
